# Effects of teriparatide on bone formation in rats with experimentally induced premaxillary expansion

**DOI:** 10.1590/2177-6709.27.3.e2220370.oar

**Published:** 2022-07-04

**Authors:** Yasin ÇAMILI, Sıddık MALKOÇ, Aslı TAŞLIDERE, Zehra İLERI, Ozge Celik GULER

**Affiliations:** 1 Private practice (Balıkesir, Turkey).; 2 Private practice (Istanbul, Turkey).; 3 İnönü University, Faculty of Medicine, Department of Histology and Embriology (Malatya, Turkey).; 4 Selçuk University, Faculty of Dentistry, Department of Orthodontics (Konya, Turkey).; 5 Çanakkale Onsekiz Mart University, Faculty of Dentistry, Department of Orthodontics (Çanakkale, Turkey).

**Keywords:** Maxillary expansion, Teriparatide, TGF-β, Osteonectin, Osteocalcin

## Abstract

**Objective:**

This study aimed to evaluate the effects of systemic teriparatide on sutural bone formation after premaxillary suture expansion in rats.

**Material and Methods:**

Twenty Wistar male rats (8-10 weeks old) were randomly divided into two groups, namely, control (C, n=10) and teriparatide (T, n=10). An expansion force was applied to the maxillary incisors using helical spring for a seven-day expansion period, for both groups. On the eighth day, the rats were kept for a seven-day consolidation period, and then 60 µg/kg teriparatide (once a day) was administered to group T subcutaneously for seven days. Then, all the rats were sacrificed, and histological sections were stained with hemotoxylin-eosin for examination. Anti-osteonectin, anti-osteocalcin, anti-Vascular endothelial growth factor (VEGF) and anti-transforming growth factor beta (TGF-β) were evaluated by immunohistochemical analysis in the midpalatal suture area.

**Results:**

Histologically, the newly formed bone tissue was observed to be larger in group T than in group C. The number of immunoreactive osteoblasts for osteonectin, osteocalcin and VEGF antibodies was significantly higher in group T than in group C (*p* = 0.0001). The TGF-β antibody showed a mild reaction in group T, but did not reach significance in comparison with group C (*p* ˃ 0.05).

**Conclusion:**

Systemic teriparatide application following the premaxillary expansion of the suture area may stimulate bone formation and add to the consolidation of the expansion in rats by regulating osteonectin, osteocalcin and VEGF.

## RESUMO

**Objetivo:**

O presente estudo teve como objetivo avaliar os efeitos do uso sistêmico da teriparatida na formação óssea sutural após a expansão da pré-maxila em ratos.

**Material e Métodos:**

Vinte ratos machos da raça Wistar (com oito a dez semanas de vida) foram divididos aleatoriamente em dois grupos: controle (C, n=10) e teriparatida (T, n=10). Uma força de expansão foi aplicada aos incisivos superiores, usando uma mola helicoidal, por um período de expansão de sete dias em ambos os grupos. No oitavo dia, os ratos iniciaram um período de sete dias de consolidação, nos quais 60 µg/kg de teriparatida foram administrados (uma vez ao dia), por via subcutânea, para o grupo T. Posteriormente, todos os ratos foram sacrificados e cortes histológicos corados com hemotolixina-eosina foram examinados. Por meio de análise imuno-histoquímica da região da sutura palatina mediana, avaliou-se a presença de anti-ostenectina, anti-osteocalcina, anti-fator de crescimento endotelial vascular (VEGF) e anti- fator transformador de crescimento (TGF-β).

**Resultados:**

Histologicamente, observou-se que o tecido ósseo recém-formado foi maior no grupo T do que no grupo C. O número de osteoblastos imunorreativos para anticorpos de osteonectina, osteocalcina e VEGF foi significativamente maior no grupo T do que no grupo C (*p* = 0,0001). O anticorpo TGF-β mostrou uma pequena reação no grupo T; porém, sem diferença significativa para o grupo C (*p* ˃ 0,05).

**Conclusão:**

O uso sistêmico de teriparatida após a expansão da sutura na região da pré-maxila pode estimular a formação óssea e melhorar a consolidação da expansão em ratos, por meio da regulação de osteonectina, osteocalcina e VEGF.

## INTRODUCTION

Expanding the midpalatal suture using rapid maxillary expansion (RME) appliances is commonly utilized in orthodontic treatment of malocclusions exhibiting a narrow maxillary jaw and posterior crossbite. The main principle of RME appliances is to rapidly expand the upper dental arch, followed by the active bone formation in the midpalatal suture.^1^ ^-^ ^3^ After RME, the stabilization of the expansion obtained is required because the upper jaw tends to revert to its previous form, despite a consolidation period.^3^ This relapse rate after RME has been reported in the literature to be as high as 63%.^4^ Many reasons have been reported to cause this relapse in the maxilla. The main reason has been suggested to be the tension in the midpalatal area and in the surrounding sutures. Nevertheless, the bone organization in the midpalatal suture completed in 3-12 months was reported to prevent this relapse.^5^ The amount of bone formed in the midpalatal suture could also shorten the period of consolidation,^3^ as previously shown in the literature.^6^

Teriparatide is a synthetic derivative of the parathyroid hormone (PTH) produced by laboratory techniques. It plays a significant role in providing the bone mineral balance of PTH, increasing the absorption of calcium from the intestines and calcium and phosphate reabsorption from the kidneys, releasing calcium from the bone to the blood according to the body’s needs, and regulating vitamin D metabolism.^7^ Systemic teriparatide was previously used to slow down osteoporosis-related bone loss and to treat fractures without cortical and trabecular bridges. It can increase osteoblastic activity by modulating the nuclear kappa B ligand/osteoprotegerin (RANKL/OPG) and the insulin-like growth factor, previously found to be involved in bone metabolism.^8^ ^-^ ^10^ Teriparatide was also found to promote bone formation during orthodontic treatments for prosthetic purposes^11^ and periodontitis-related bone loss.^12^

Biochemical markers are indicative of complex bone remodelling processes. Osteocalcin is a pro-osteoblastic non-collagen protein found in bone and dentin, and it plays a role in bone building and metabolic regulation.^13^ Its expression is also induced by teriparatide.^14^ ^-^ ^16^ Osteonectin, a non-structural matricellular glycoprotein secreted by osteoblasts, binds calcium in bone^17^ and may also be increased by teriparatide.^18^ ^,^ ^19^ VEGF^20^ and TGF-β^13^ are involved in tissue regeneration and reproductive functions activated by teriparatide.^21^

Several experimental studies evaluated the effects of different agents, such as resveratrol, propolis and lithium, on bone formation in expanded sutures.^22^ ^-^ ^24^ However, according to our knowledge, no study has evaluated the efficiency of teriparatide on bone formation in experimentally expanded sutures. Given the beneficial effects of teriparatide on osteoblastic activity and the origin of teriparatide as a synthetic derivative of a hormone produced by the human body, this study aimed to evaluate the effects of systemic teriparatide application on the bone formation of premaxillary suture in rats treated with maxillary expansion.

## MATERIALS AND METHODS

### ANIMALS AND STUDY GROUPS

Twenty 8-10-week-old male Wistar rats weighing 200±15 g were randomly and equally divided into two groups: group T (n=10), which was treated with teriparatide following the placement of the expansion appliance; and group C (n=10), which served as the healthy control group with only the expansion appliance and without teriparatide administration. During the experimental period, all the rats were kept in separate cages in a quiet room with a controlled temperature (23°C) and a 12 h light and dark cycle. The rats were fed a standard and solid diet, and had access to tap water *ad libitum*. After appliance placement, they were fed with a softened rat diet, in order not to use their teeth and break the appliance. The experimental protocol was approved by the Ethical Committee on Animal Research of İnönü University (2013/A-49) and conducted in the Department of Experimental Animals, Research and Application Centre.

The sample size for each group was calculated on the basis of an alpha significance level of 0.05 to achieve 90% power to detect significant differences with a 0.40 effect size (G*Power version 3.0.10; Franz Faul Universidad, Kiel, Germany).^23^ The power analysis showed that 20 samples in the parallel group were required, and animals were randomly divided into two groups.

### APPLIANCE PLACEMENT

**Figure 1:**
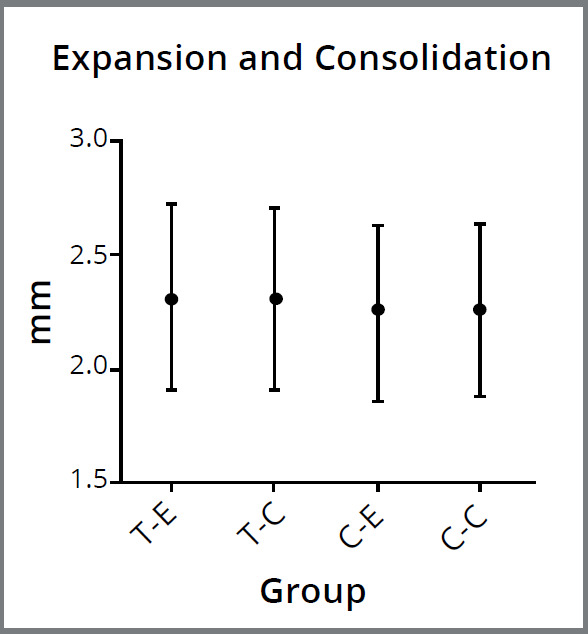
Measured values between the mesial edges of the two incisors of the subject after the expansion period (E) and the consolidation period (C). The Mann-Whitney U and Friedman tests were used for statistical analysis. No difference was found for the distance (*p*˃ 0.05). T-E = Teriparatide group-Expansion period; T-C = Teriparatide group-Consolidation period; C-E = Control group-Expansion period; C-C = Control group-Consolidation period).

The rats were checked by a veterinarian, who confirmed that their general health conditions were normal. The animals were weighed for weight change before and after the experiment. The upper incisors of the rat were preferred because of their ease of application and their usability to simulate the maxillary expansion in previous studies.^1^ ^,^ ^5^ ^,^ ^6^ ^,^ ^23^ In the maxilla of each rat, the distance between the mesial edges of the two incisors of the rats was measured in the cervical level twice with a caliper (Fig 1), after the expansion (T1) and after the consolidation (T2), initially assumed to be 0 mm (T0).

**Figure 2:**
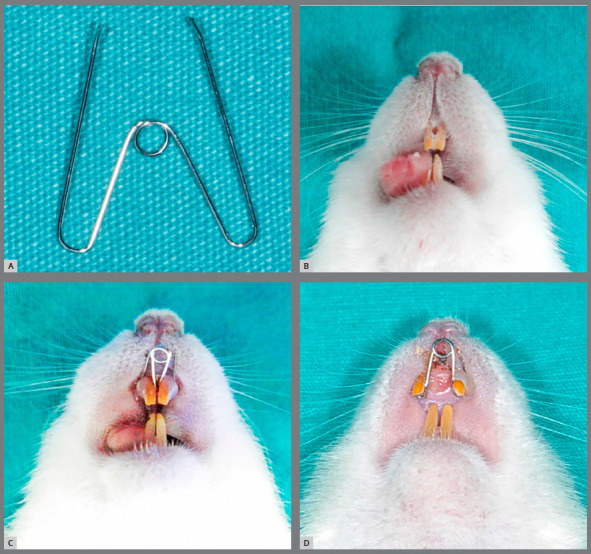
Image of the expansion spring (A), the retention regions of upper incisors for appliance (B), appearance of the appliance in the mouth (C), after expansion and opening of the suture (D).

The animals were anesthetized with a combination of xylasine (5 mg/kg intraperitoneally; Alfazyne-Alfasan International B.V. Woerden, Netherlands) and ketamine hydrochloride (50 mg/kg intraperitoneally; Alfazyne-Alfasan International B.V. Woerden, Netherlands). A helical spring fabricated from a 0.014-in stainless steel wire (Dentaurum, Ispringen, Germany) used for the midpalatal suture expansion was positioned in the retention regions drilled close to the gingival margins of both upper incisors on the mesial side (Figs 2A and 2B). A composite adhesive (Transbond XT, Seefeld, Germany) was used circularly to the helicals, to increase the retention of the springs (Fig 2C). The springs were placed on a grid and activated on a single arm with a plier. Then they were adjusted with a force gauge (Dentaurum, Ispringen Germany) to give 1.176 N^25^ when placed on the mesial of the incisors, and were not reactivated during the seven-day expansion period.^3^

After the expansion period, the experiment was conducted with a seven-day consolidation period. At the end of the expansion period, the springs were removed and controlled to be passive and then replaced as a retainer during the retention (Fig 2D). After the consolidation, the distance between the mesial edges of the upper incisors was measured with a manual caliper (Dentaurum, Ispringen, Germany).^22^ This distance was also measured after the expansion period.

### TERIPARATIDE APPLICATION

Teriparatide is only available in one form (Forsteo^®^, Eli and Lilly Company, Indiana, USA) in the market. According to the manufacturer’s protocol, during the seven-day consolidation period, 60 µg/kg daily teriparatide^26^ ^,^ ^27^ was injected subcutaneously into the animals of group T. The animals in group C were injected subcutaneously physiological saline (60 µg/kg, 0.9% NaCl) during the consolidation period.

### HISTOLOGIC ANALYSIS

All the rats were sacrificed with 200 mg per kilogram of sodium pentobarbital (Pentothal, Abbot, North Chicago, Ill). The premaxillae were resected as a block and fixed in 10% formalin. Then, the retaining wires were removed, and the premaxillae were decalcified with tetraacetic acid for three weeks. After decalcification, the premaxillae were cut into parts containing two incisor teeth. These incisors served as the primary guide for orienting the sections. Two points were chosen after cutting the section to the sagittal plane perpendicularly. The first one was located at the alveolar crest, and the second one was located 4 mm apical to the crest. This imaginary line passed through the center of the incisor crown at its gingival portion.^22^ The paraffin blocks were sectioned serially at 5-µm intervals, and the orientating sections were stained with hematoxylin and eosin (H&E; Harris haematoxylin, 09-182-1; DDK Italia, Italy; Eosin, MOS, 0712012, Turkey) after the histopathological examination for deparaffinization and rehydration. The sections were examined under a light microscope (Leica DFC 280) in the upper jaw suture region for the new bone formation area.

### IMMUNOHISTOCHEMICAL ANALYSIS

The indirect immunoperoxidase method was used for immunohistochemical staining. After deparaffinization and incubation sessions, the sections were washed in distilled water and phosphate-buffered saline for 10 min, respectively, and incubated with a non-immune serum (Ultra V block, cat. No:TA-125-UD, Lab Vision) prior to incubation with the primary antibodies anti-osteonectin (SPARC, 251503, Abbiotec, San Diego, CA, USA), anti-osteocalcin (OC4-30, Novus Biologicals, Cambridge, UK), anti-VEGF (PU483-UP, Biogenex, San Ramon, CA, USA) and anti-TGF-β (ab66043, Abcam plc, Cambridge, UK) for 18 h at 4°C. After washing, the secondary staining protocol was performed, and the sections were kept in streptavidin peroxidase (Lab. Vision CAT: TS-125-HR) for 20 min. Finally, the sections were mounted on an aqueous mounting medium and sent to İnönü University School of Medicine Department of Histology for evaluation. After the staining sections were examined under a light microscope, the number of active osteoblasts was scored as +(1-10), ++(11-20) and +++(˃20) in 40× magnification. Then, the sections were examined with a light microscope (Leica DFC 280, Cambridge, UK) and analyzed with the Leica Q win system (Breckland, UK). A red-brown precipitate indicated positive findings for the primary antibodies. A single examiner, blinded to the clinical procedures, performed the evaluations, and the results were taken as the average of the counts. The measurement procedures were repeated after four weeks by the same investigator. For all measurements, the intraclass correlation coefficient was 0.907-0.980, which means that acceptable reliability and reproducibility of all measurements was achieved.

## STATISTICAL ANALYSIS

Statistical analysis of the distance between the teeth and the weight follow-up data on 20 subjects with different histological and immunohistochemical data was performed by SPSS 22.00 (SPSS Inc. Chicago, Illinois, USA). The Shapiro-Wilk test was used as a normality distribution test. The Mann-Whitney U test was used to determine the differences between groups of data obtained from the measurements of the distance between the mesial edges of the two incisors at different measurement times until the end of the consolidation period. The Friedman test was applied to the group evaluations to assess the difference in weight change between the baseline and before sacrifice. The Mann-Whitney U test was used for the intergroup evaluation. The immunohistochemical data were compared among the subjects using the Mann-Whitney U test after the consolidation period, and the error level was taken as α = 0.05.

## RESULTS

At the beginning of the study, 24 Wistar rats were included, but three rats were lost after general anaesthesia. The remaining 21 rats were observed to tolerate the expansion well during expansion and consolidation. No abnormal findings, in terms of general health conditions, were found in the weight measurements made at the beginning and at the end of the experiment. On the sixth day of the consolidation phase, one eye infection was observed in a rat, which was removed from the study. Finally, the study was completed with 20 rats (10 rats in each group).

**Figure 3:**
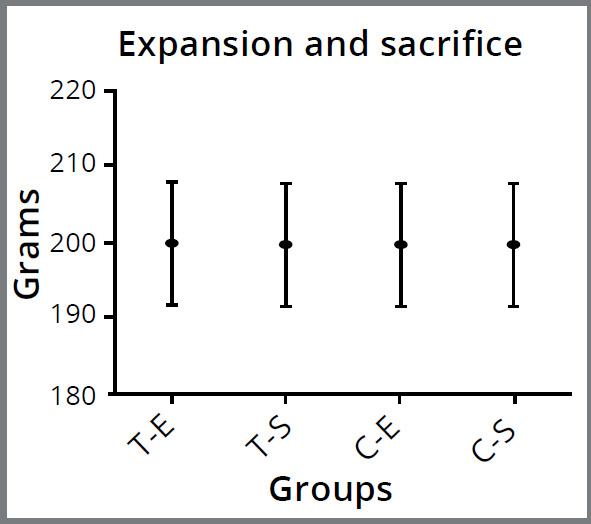
Body weights before expansion (E) and before sacrifice (S) of the groups. The Mann-Whitney U and Friedman tests were used for statistical analysis. No difference was found in body weights (*p* ˃ 0.05). T-E = Teriparatide group-Expansion period; T-S = Teriparatide group-Sacrifice period; C-E = Control group-Expansion period; C-S = Control group- Sacrifice period).

No statistically significant difference was found in the comparison of body weights within and between groups before the experiment and before being sacrificed (*p* > 0.05) (Fig 3). No significant difference was observed within and between groups for the distance between the mesial edges of the teeth at the end of the expansion protocol and before sacrifice (*p* > 0.05) (Fig 1).

### HISTOLOGICAL AND IMMUNOHISTOCHEMICAL FINDINGS

**Figure 4:**
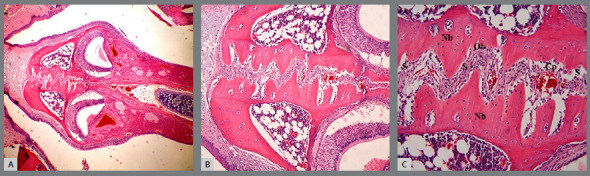
Midpalatal suture photographs obtained in Group T. T = Teriparatide group, S = Suture region, Nb = New bone region, Ob = Osteoblast, Cp = Capillary. A) H-E; X4, B) H-E; X10, C) H-E; X20.

**Figure 5:**
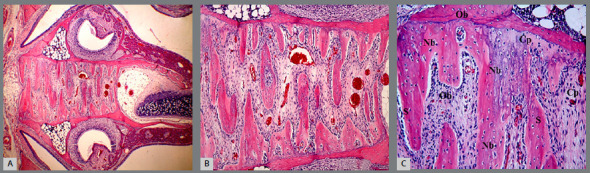
Midpalatal suture photographs obtained in Group C. C = Control group, S = Suture region, Nb = New bone region, Ob = Osteoblast, Cp = Capillary. A) H-E; X4, B) H-E; X10, C) H-E; X20.

All sections stained with H&E were examined in the upper jaw suture region under a light microscope. The new bone formation was observed in group T (Fig 4) at a more intense level than that in group C (Fig 5) in visual evaluation. In the suture of group C, new bone areas were observed in the form of islands and extensions. Conversely, in group T, bone formation was shown as merged wide areas.

**Figure 6:**
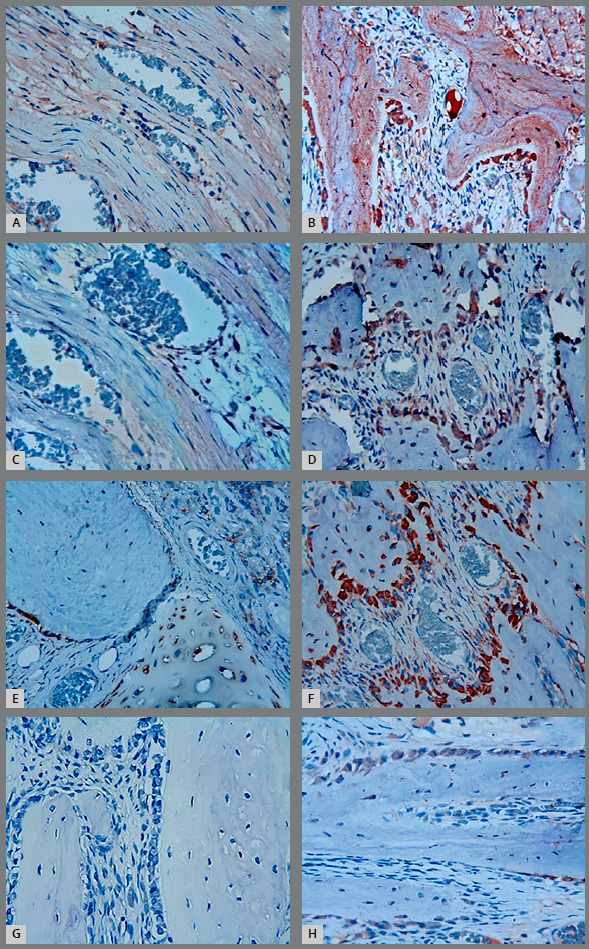
Immunohistochemical staining of the suture specimens. Osteocalcin (**A**- Group C, **B**- Group T), Osteonectin (**C**- Group C, **D**- Group T), VEGF (**E**- Group C, **F**- Group T) and TGF- β (**G**- Group C, **H**- Group T). Except for TGF-β, positive stained brown cells were observed in Group T. There were no positive stained cells in the suture zone of Group C, X40. T = Teriparatide group, C = Control group.

The results of osteonectin, osteocalcin, VEGF and TGF-β immunoreactivity of the immunohistochemically stained sections are shown in Figure 6. In group T, indirect immunohistochemical staining with osteocalcin (Figs 6A, B), osteonectin (Figs 6C, D) and VEGF (Figs 6E, F) showed high immunoreactivity in the osteoblasts, but a slight increase in TGF-β (Figs. 6G, H) was observed. In group C, mild immunoreactivity was observed in the osteoblasts in the evaluation of the indirect immunohistochemical staining with osteocalcin, osteonectin, VEGF and TGF-β antibodies (Figs 6A, C, E and G).

**Figure 7:**
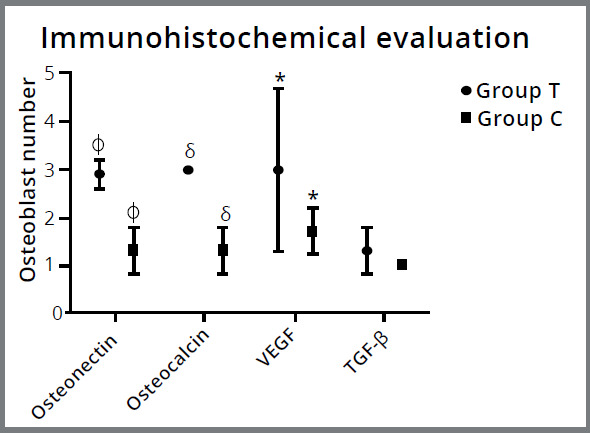
Immunohistochemical evaluations. The Mann-Whitney U test was used for statistical analysis. Osteonectin, osteocalcin and VEGF antibodies were significantly higher in group T than in group C (*p* ^ϕ,δ,*^ = 0.0001), but no differences were found in TGF-β antibody (*p* ˃ 0.05) for osteoblastic immunoreactivity. T = Teriparatide group, C = Control group.

A statistically significant difference was found in the osteoblastic immunoreactivity for osteonectin, osteocalcin and VEGF antibodies in group T, in comparison with group C (*p* = 0.0001) (Fig 7). No statistically significant difference was found between the groups for TGF-β antibody osteoblastic immunoreactivity (*p* ˃ 0.05; Fig 7), but a slight antibody staining was observed in group T that did not reach significance (Fig 6H).

## DISCUSSION

Teriparatide, a derivate of PTH, has been used for the treatment of several bone-related diseases such as osteoporosis, because of its superiority in bone formation and anabolic activity.^7^ To the best of our knowledge, no study has evaluated the effects of teriparatide on bone formation after maxillary expansion. Therefore, this study aimed to evaluate the effects of teriparatide on bone formation in rats around the midpalatal suture region after maxillary expansion, with the aim of preventing relapse in the consolidation period. According to the results, teriparatide administration was found to increase osteoblastic activity in the midpalatal suture region of rats by upregulating osteonectin, osteocalcin and VEGF.

In the literature, several experimental studies^26^ ^,^ ^28^ ^-^ ^30^ evaluated the effect of teriparatide with different doses on bone healing and formation in rats, and reported that teriparatide increased bone healing and formation independent of the dose. They found that a low dose of 5-60 µg/kg was more effective. Seebach et al.^31^ evaluated the effect of teriparatide with a dose of 60 µg/kg on rats treated with distraction osteogenesis, and reported that teriparatide accelerated bone formation during consolidation, and improved mechanical strength. Some researchers^32^ defined the RME as a type of distraction osteogenesis, as both techniques aim to create bone formation. Thus, in the current study, we used the same dose of 60 µg/kg similar to Seebach et al,^31^ and observed an accelerated bone formation during the consolidation period, histologically and histochemically.

Teriparatide has been used for different purposes in dentistry. Shirota et al.^33^, Ohkawa et al.^34^, Aggarwal and Zavras^11^ reported that teriparatide reduced bone loss after implantation and caused improved osseointegration. Marques et al.^12^ and Bashutski et al.^35^ examined the effects of intermittent teriparatide on periodontitis, and reported that teriparatide prevented bone loss due to periodontitis and allowed a better healing of the defect. The effects of teriparatide on tooth movement during orthodontic therapy were evaluated by Salazar et al,^36^ who examined the effects of subcutaneous intermittent teriparatide (30 µg/kg) on the tooth movement of the maxillary first molars in ovariectomized rats for 90 days, and concluded that the rate of orthodontically induced tooth movement was greater in rats treated with teriparatide, because of its activation effects on osteoblasts. They^36^ reported that there was more tooth movement when injected teriparatide, without a significant increase in osteoclast number, since teriparatide normalized bone density. In another study, Soma et al.^37^ reported that the continuous application of 10 µg/kg teriparatide for 7 days could accelerate tooth movement. Soma et al.^38^ examined the effects of local and chronic PTH (I-34) dissolved in methylcellulose gel on the experimental tooth movement in rats, and found no inhibitory effect on orthodontic tooth movement. Soma et al.^38^ stated that intermittent systemic injection of teriparatide stimulated bone formation without osteoclastic effect, but the continuous infusion of teriparatide induced both bone formation and resorption, without a net reduction in the bone mass. In this study, teriparatide was found to be beneficial to bone formation after the maxillary expansion of the midpalatal suture. However, the effect of teriparatide on tooth movement was not evaluated, and thus a full comparison of the present study with the literature was not made. Therefore, conducting further studies with an experimental design thath sets up both the premaxillary expansion in the upper incisors and tooth movement in the maxillary molars, may allow the comparison. The relapse after RME depends on many factors due to bone metabolism and stress in the midpalatal and circummaxillary sutures. Circummaxillary suture system with age is a very critical determinant of relapse. Since this study only investigated the effect on midpalatal suture, further studies are needed to examine circummaxillary sutures.

Studies investigating the effects of teriparatide used a variety of methods, including histologic,^12^ ^,^ ^13^ histomorphometric,^34^ radiological^11^ ^,^ ^12^ and tomographic^11^ methods. However, to the best of our knowledge, no study has yet investigated the effects of teriparatide on bone formation by the immunohistochemical method, which may provide reliable and reproducible results, because of the targeting of a smaller and more specific structure of cells. According to the present results, the immunoreactivity of the osteoblasts for osteonectin, osteocalcin and VEGF antibodies in the suture region was higher in group T than in group C (*p* = 0.0001). Histologically, the excess of the number of osteoblasts in the suture zone in group T was consistent with the finding that the amount of newly formed bone in the suture region is higher than that in group C. In group T, the increase in the number of osteoblasts resulting from the increase in osteoblast stimulation was similar to previous studies.^1^ ^,^ ^6^ ^,^ ^22^ ^,^ ^39^

Birlik et al.^13^ evaluated the effects of sex steroids, namely, testosterone and oestrogen, on bone formation following a midpalatal suture expansion in rats. The female experimental group was given 17b-estradiol at a dose of 0.1 mg/kg daily, and the male experimental group was administered testosterone at a dose of 4 mg/kg intramuscularly throughout the expansion and retention periods of 7 and 5 days, respectively. The authors^13^ detected a strong immunoreactivity of VEGF, TGF-β, osteocalcin and osteonectin in the osteoblasts and in the connective tissues of sutures. In the current study, indirect immunohistochemical staining with osteocalcin, osteonectin and VEGF showed a high immunoreactivity in the osteoblasts, but the slight increase in TGF-β immunoreactivity in group T did not reach significance. However, a full comparison was not made because of the differences in the study designs of Birlik et al.^13^ (orchiectomised and ovariectomised rats) and the present study, and in the retention times. 

After the maxillary expansion procedures, different retention periods such as 7,^3^ ^,^ ^39^ 10^40^ and 12 days^41^ were used. However, no consensus has been made in the literature on the retention period required after RME. The response after orthodontic force is about 30-40 hours in humans and 6 hours in rats, and the basic mechanisms in these changes are similar to humans.^42^ Saito and Shimizu^3^ found that determining the bone formation that would occur in a 3 day retention period would be difficult, and that applying it for 7 days would be sufficient. Thus, we used the same retention protocol, including the 7 days. Similar to previous studies,^3^ ^,^ ^19^ ^,^ ^24^ ^,^ ^39^ the midpalatal suture was examined in the current study for suture ossification because it is supported by the incisor teeth for sutural separation without any surgical operation, thus reducing the risk of infection and additional cost. In the present study, a modified appliance of Saito and Shimizu ^3^ was used to provide sutural separation with incisor teeth support and to prevent difficulties during the placement of wires around the spring due to the small volume of the rat maxilla. Furthermore, we used systemically administration of teriparatide^27^ ^,^ ^28^ rather than a local injection, as suggested in the literature, during orthodontic treatment, because of the possibility of a direct effect on the osteoblasts.^43^ Local administration was reported to fail because it could not specifically target the osteoblastic activity.^3^ ^,^ ^39^ The ease of application, the vascular structure of the maxilla, and the wide use of systemic administration in the literature makes it a more effective method than local injection. However, systemic application was claimed to be involved in jaw bone necrosis.^44^ In addition, systemic usage was found to cause bone tumors by using a 30 µg/kg daily dosage for 2-6 months in rats.^45^ In the current study, no abnormalities from bone analysis were detected. Side effects such as headache, nausea, osteoporosis, fatigue and depression were reported to occur as a result of the continuous use of systemic teriparatide.^11^ Some studies emphasized the need for caution on the use of teriparatide in individuals with chronic diseases, such as osteosarcoma, Paget’s disease, hypercalcemia and bone metastasis. Thus, local administration in chronic disease was found to have fewer side effects.^46^

The effects of teriparatide administration were determined in animals. Therefore, more extensive studies on humans, comparing local and systemic administration, are needed. The other limitations of this study include the lack of groups with different doses and time periods of teriparatide administration, and the lack of additional biochemical markers involved in bone turnover, such as bone alkaline phosphatase and tartrate-resistant acid phosphatase 5b. Further studies to confirm the beneficial effects of teriparatide usage after maxillary expansion treatment are required.

## CONCLUSION

Teriparatide administration can be used to facilitate stability during consolidation by stimulating new bone formation after an experimentally induced maxillary expansion, by regulating osteonectin, osteocalcin and VEGF.
